# Citizen satisfaction with public services in Peru: Regional gaps in citizen perceptions of public service provision

**DOI:** 10.1371/journal.pone.0349632

**Published:** 2026-06-12

**Authors:** Miluska Odely Rodriguez-Saavedra, Iván Cuentas Galindo, Luis Miguel Campos Ascuña, Antonio Víctor Morales Gonzales, José Luis Chavez Cuarite, Adolfo Erick Donayre Sarolli, Erick Alexander Donayre Prado, Robinson Bernardino Almanza Cabe, Ruben Washington Arguedas Catasi

**Affiliations:** Faculty of Economics, Universidad Nacional de San Agustín de Arequipa, Arequipa, Perú; University of Pretoria, SOUTH AFRICA

## Abstract

Citizen satisfaction with public services is a fundamental indicator of government effectiveness and democratic legitimacy in Latin America. However, there is little empirical evidence on regional gaps in countries with high territorial heterogeneity such as Peru. The objective of this study was to determine the differences in levels of citizen satisfaction with public services between the country’s coastal, mountain, and jungle regions. A secondary analysis was conducted of the 2024 National Household Survey, corresponding to the Governance, Democracy, and Transparency Module (n = 33,691), evaluating 21 public services using the Citizen Satisfaction Index. Student’s t-tests were applied for comparisons between urban and rural areas, one-factor ANOVA with Bonferroni post-hoc tests for comparisons between geographic domains, and principal component analysis to explore the dimensional structure. The results revealed statistically significant differences between the eight geographic domains (F = 89.57, p < .001, η² = .022); although this effect size is small, indicating that geographic domain accounts for 2.2% of the variance in satisfaction, the patterns are consistent and policy-relevant. The North Coast (M = 3.09) and Amazon (M = 3.13) showed the lowest satisfaction rates, while the Southern Highlands (M = 3.29) even surpassed Metropolitan Lima. Social programs (51.9%) and the Public Prosecutor’s Office (56.4%) obtained the lowest national satisfaction ratings, with the largest regional gaps. Rural areas reported higher satisfaction with public transportation (+7.0%) and public education (+3.5%) than urban areas. A four-dimensional factor structure was identified that explains 46.7% of the variance. It is concluded that territorial gaps are consistent with structural differences in regional institutional capacity, requiring territorially differentiated public policies to reduce inequalities in the provision of state services.

## 1. Introduction

Citizen satisfaction with public services is a key indicator for assessing government effectiveness and democratic legitimacy in Latin America [1]. In decentralized systems, citizen perceptions of service provision reflect both the administrative capacity of the state and the structural inequalities that characterize emerging economies [2]. In this regard, Peru has significant territorial disparities between its coastal, Andean, and Amazonian regions, where differences in economic development, infrastructure, and institutional capacity generate marked gaps in access to and quality of basic services such as health, education, and public administration. Since the implementation of administrative decentralization in 2002, responsibilities for the provision of public services have been progressively transferred to regional and local governments, creating a scenario where subnational institutional quality directly determines citizens’ experience with state services. More than two decades into this process, a cross-sectional snapshot of citizen satisfaction in 2024 provides relevant evidence on the current state of territorial equity in service provision.

International research identifies multiple determinants of citizen satisfaction with public services. At the institutional level, the quality of local governance and administrative capacity determine the effectiveness of service provision [3,4], while innovation in public management and accountability mechanisms significantly improve citizen perception [5]. Likewise, at the territorial level, urban-rural disparities in infrastructure and specialized human resources generate systematic gaps in access to essential services [6]. For example, quantitative studies in Ecuador document that rural citizens report 23% lower levels of satisfaction compared to urban residents in health services and public administration [7].

Similarly, recent empirical evidence in emerging economies reveals that regional gaps persist even after formal administrative decentralization processes. In Indonesia, analyses of 61 smart cities show that the implementation of e-government did not reduce disparities between central and peripheral regions due to persistent differences in local institutional capacity [8]. On the other hand, in Vietnam, multi-province research identifies factors such as specialized human capital, physical infrastructure, and local fiscal resources as accounting for 67% of the variation observed in citizen satisfaction [9]. Consequently, longitudinal studies in Honduras find that decentralization improved satisfaction only in municipalities with previously robust institutional capacity, while localities with weak institutions experienced a deterioration in perceived quality [10].

However, there is a substantial gap in knowledge about regional gaps in citizen satisfaction with public services in Peru. Despite the relevance of the issue, the available research is limited to single case studies, such as the analysis of satisfaction with vaccination services in Lima during the COVID-19 pandemic [11], without addressing interregional differences or other essential public service sectors. Furthermore, there are no systematic comparative analyses that quantify differences in citizen satisfaction between the country’s geographically diverse regions, nor are there any studies that examine how local institutional characteristics are associated with these perceptions. This lack of empirical evidence is critical considering that Peru exhibits higher levels of territorial heterogeneity than other Andean countries, with coastal regions that have human development indicators comparable to upper-middle-income nations, while areas in the highlands and jungle remain similar to low-income countries. Therefore, the absence of rigorous quantitative diagnoses limits the design of differentiated public policies that strengthen decentralized governance and reduce territorial inequalities in the provision of state services.

Within this framework, the overall objective of this research is to determine the differences in levels of citizen satisfaction with public services between selected regions of the coast, highlands, and jungle of Peru. The specific objectives are: i) to compare levels of citizen satisfaction between the regions studied according to the type of public service evaluated, ii) to examine differences in citizen satisfaction between urban and rural areas, iii) to identify the services with the greatest regional gaps in satisfaction according to their institutional complexity, and iv) to explore the dimensional structure of citizen satisfaction with public services.

This study makes three contributions to scientific knowledge. First, it provides theoretical evidence on how institutional and territorial variables are associated with citizen satisfaction in a highly decentralized system with marked regional heterogeneity, extending previous findings by Naveen and Sharma [12] and Din et al. [13] to economies with greater territorial inequalities. Second, it empirically offers the first quantitative comparative characterization of citizen satisfaction among geographically differentiated regions in Peru, filling a critical gap in the literature on decentralized governance in Latin America. Finally, from a practical perspective, it generates specific and quantifiable evidence that allows public policy designers to identify critical dimensions of service quality that require priority intervention in each type of region, facilitating the efficient allocation of resources to reduce territorial gaps in the provision of state services and contributing to the fulfillment of Sustainable Development Goal 10 on reducing inequalities within countries.

## 2. Literature review

Citizen satisfaction with public services is a well-established field in public administration, with growing attention to territorial disparities in developing countries. This review examines the theoretical frameworks of citizen satisfaction, the role of institutional quality in decentralized contexts, evidence on territorial gaps, and knowledge gaps, providing a basis for research hypotheses.

### 2.1 Theoretical foundations of citizen satisfaction

The expectation disconfirmation model is the dominant conceptual framework for understanding citizen satisfaction with public services. This model establishes that citizens evaluate perceived quality by comparing it with their prior expectations, generating satisfaction when performance exceeds expectations and dissatisfaction when it falls short. Moreno-Menéndez et al. [14] demonstrated the cross-cultural validity of this model through meta-analysis, establishing that the gap between expectations and perceived performance is the most consistent predictor of citizen satisfaction. Radika et al. [15] showed that institutional quality standards shape citizen expectations, explaining why regions with objectively similar services may have different levels of satisfaction.

Institutionalist theory emphasizes that the institutional environment determines both the capacity for provision and citizens’ perceptions of state legitimacy. Song et al. [16] demonstrated that institutional quality influences trust in state organizations regardless of available material resources. In this study, institutional quality is understood as the structural capacity of public organizations to deliver services effectively, accountably, and equitably across territories. Citizen satisfaction is treated as a perceptual outcome associated with this capacity rather than a direct measure of it: satisfaction reflects how citizens experience and evaluate service provision, which is shaped by both objective institutional performance and subjective expectations calibrated to local conditions. In emerging economies, the quality of local governance directly conditions the effectiveness of service provision, generating virtuous or vicious circles depending on pre-existing administrative capacity.

### 2.2 Institutional quality and decentralization processes

Institutional quality acts as a mediating variable between service provision and citizen perception. Rico et al. [17] demonstrated that the regional institutional environment explains significant differences in satisfaction even when controlling for budgetary variables, while Hai et al. [18] found that effective local institutional arrangements reduce corruption risks and improve service provision. Collaborative governance between levels of government emerges as a particularly relevant factor for perceived quality.

Administrative decentralization processes generate mixed results depending on prior institutional capacity. Kurti and Kina [19] documented that decentralization improved satisfaction only in municipalities with robust institutional capacity, while localities with weak institutions experienced deterioration. Metwally and Samir [20] demonstrated that the implementation of e-government reforms did not reduce disparities between central and peripheral regions due to persistent differences in local institutional capacity. These findings suggest that decentralization reforms can amplify preexisting territorial inequalities in the absence of simultaneous institutional strengthening.

International comparative evidence reveals that territorial disparities in satisfaction persist even in countries with relatively high levels of institutional development. Pribadi [21] documented significant variations between provinces, attributing these differences to local fiscal capacity and the availability of specialized human capital. Zhang et al. [22] demonstrated in the Latin American context that perceived quality predicts satisfaction and that this generates loyalty to local government, suggesting that satisfaction with specific services can generate spillover effects toward overall government legitimacy.

### 2.3 Territorial disparities and differentiation by context

Urban-rural gaps are a recurring manifestation of territorial disparities in citizen satisfaction. Ochoa- Gabinete et al. [23] found that rural residents consistently report lower satisfaction with health, education, and security services, although paradoxically they report higher satisfaction with public transportation. Kim et al. [24] attributed these disparities to limited coverage, long response times, and less trained staff in remote areas, although they also documented that rural satisfaction can exceed urban satisfaction in services where expectations are proportionally lower.

Predictors of satisfaction vary substantially depending on the type of service and the institutional complexity required. Cohen et al. [25] identified that quality dimensions such as reliability, responsiveness, and empathy are stronger predictors than tangible elements in complex administrative services, while Prokop and Tepe [26] reported that accessibility and speed emerge as critical dimensions in basic urban services.

Research on sociodemographic determinants yields mixed results. Reddick et al. [27] documented a positive association between age and satisfaction, interpreting this pattern as a result of generational differences in expectations. With regard to gender, the evidence is contradictory: while some studies find differences that disappear when controlling for income, others show that political representation is positively associated with citizen satisfaction.

### 2.4 Gaps in scientific knowledge

There are substantial gaps in knowledge about regional disparities in citizen satisfaction with public services in emerging economies with high territorial heterogeneity. The available research focuses on single case studies or contexts in developed countries. Li et al. [28] documented substantial improvements in satisfaction through specific management interventions in vaccination services, demonstrating that institutional changes can generate significant changes. However, these findings do not address systematic interregional differences or allow for generalization to other sectors [29].

There are no comparative analyses that quantify differences in satisfaction between geographically diverse regions in contexts of extreme territorial heterogeneity. This absence is critical for contexts where regions have dramatically divergent levels of institutional and economic development. The institutional architecture of decentralized systems assigns differentiated responsibilities between levels of government, suggesting that satisfaction can be structured dimensionally according to type of service, a hypothesis that remains without systematic empirical evaluation.

The absence of rigorous quantitative diagnoses limits the design of territorially differentiated public policies. The international literature suggests that the effects of decentralization depend critically on prior institutional capacity, raising the question of whether patterns observed in Asian and Central American economies are replicated in Latin American contexts with greater structural territorial inequality.

### 2.5 Research hypotheses

Based on the theoretical and empirical evidence reviewed, and considering the gaps identified in knowledge about territorial disparities in citizen satisfaction in contexts of high institutional heterogeneity, the following research hypotheses are formulated:

H1: There are significant differences in levels of citizen satisfaction with public services between geographical domains.

H2: There are significant differences in satisfaction with public services between urban and rural areas, moderated by the type of service evaluated.

H3: Services that require greater institutional capacity and intergovernmental coordination present greater regional satisfaction gaps than services provided through simple decentralization.

H4: Citizen satisfaction with public services has a multidimensional structure that reflects the institutional architecture of decentralized systems.

## 3. Methodology

### 3.1 Research design

The study was conducted using a quantitative approach, with a non-experimental, cross-sectional design and a descriptive comparative scope. A secondary analysis was performed using the 2024 National Household Survey, corresponding to the Governance, Democracy, and Transparency Module. The unit of analysis consisted of citizens over the age of eighteen. Likewise, the central variable was operationalized using the Citizen Satisfaction Index, constructed with twenty-one items related to public services.

### 3.2 Population and sample

The target population consisted of citizens over the age of eighteen residing in private homes. The effective sample included 33,691 respondents distributed across eight geographical areas. The analysis incorporated the survey’s sample design, using expansion factors, strata, and clusters. Missing values were recorded in specific variables due to non-response, which reduced the number of valid cases in some descriptive sections. Missing data ranged from 18.2% for the Fire Department to 35.3% for the Comptroller’s Office depending on the service evaluated, reflecting item-level non-response rather than unit non-response. The missing data pattern was predominantly item-level, corresponding to respondents who had not used or had no opinion about a specific service. Listwise deletion was applied for each analysis, retaining all valid cases per item. To assess potential bias, results were compared between the full analytic sample and the complete-case subsample, and no substantive differences were observed in the direction or magnitude of the findings. Table 1 presents the sociodemographic characteristics of the sample.

**Table 1:** Sociodemographic characteristics of the sample.

Variable	n	%	95% CI
Gender (n = 29,546)			
Male	12,873	43.6	[43.0, 44.2]
Female	16,673	56.4	[55.8, 57.0]
Area of residence (n = 33,691)			
Urban	22,031	65.4	[64.9, 65.9]
Rural	11,660	34.6	[34.1, 35.1]
Geographic domain (n = 33,691)			
North Coast	4,751	14.1	[13.7, 14.5]
Central Coast	3,287	9.8	[9.5, 10.1]
Southern Coast	2,283	6.8	[6.5, 7.1]
Northern Highlands	2,015	6.0	[5.7, 6.3]
Central Highlands	5,531	16.4	[16.0, 16.8]
Southern Highlands	4,523	13.4	[13.0, 13.8]
Amazon	7,211	21.4	[21.0, 21.8]
Metropolitan Lima	4,090	12.1	[11.8, 12.4]

*Note: CI = 95% confidence interval. Source: ENAHO 2024, Governance Module – INEI*.

### 3.3 Study variables

The dependent variable is citizen satisfaction with public services, operationalized using a Citizen Satisfaction Index derived from 21 items organized into four dimensions. The main independent variable is geographic domain with 8 categories. Gender and area of residence were included as control variables. Table 2 presents the detailed operationalization.

**Table 2:** Operationalization of study variables.

Variable	Dimension	Indicators	Scale	Code
Dependent variable: Citizen satisfaction (ISC)	Basic services	Education, health, water, electricity, internet	Ordinal 1–4	P1$01–05,09
	Justice and security	Judiciary, Public Prosecutor’s Office, PNP, security	Ordinal 1–4	P1$06,11,16,17
	Administrative services	RENIEC, SUNARP, SAT, INDECOPI, Comptroller’s Office	Ordinal 1–4	P1$12,15,18–21
	Subnational governments	Municipalities, social programs, transportation, pensions	Ordinal 1–4	P1$03,07,10,14
Independent variable: Geographic domain	Natural region	Coast (N, C, S), Highlands (N, C, S), Amazon, Metropolitan Lima	Nominal 8 cat.	DOMAIN
Control variable: Gender	Biological sex	Male/ Female	Nominal 2 cat.	P207
Control variable: Area of residence	Geographical area	Urban/ Rural	Nominal 2 cat.	AREA

*Note. CSI = Citizen Satisfaction Index. AREA = urban/rural residence indicator derived from the ENAHO sampling frame; although labeled STRATA in the raw microdata, this variable functions as a residence area indicator, not a sampling stratum identifier.*

### 3.4 Instrument and reliability

The ENAHO.01B Questionnaire from the Governance Module, a standardized INEI instrument administered annually since 2002, was used. The module assesses satisfaction with 21 public services using a 4-point Likert scale. For the reliability analysis, only respondents with complete data across all 21 items were included, corresponding to 18,426 valid cases. Table 3 presents the reliability indicators.

**Table 3:** Reliability analysis of the instrument.

Dimension	Items	rᵢₜ	α	95% CI
Basic services	6	.52 −.68	.847	[.842,.852]
Justice and security institutions	5	.58 −.74	.891	[.887,.895]
Administrative services	7	.49 −.65	.823	[.817,.829]
Subnational governments and programs	3	.45 −.58	.756	[.748,.764]
Total scale (ISC)	21	.41 −.74	.941	[.939,.943]

*Note. n = 18,426 valid cases. rᵢₜ = corrected item-total correlations. α = Cronbach’s alpha. Criteria: α ≥ .90 excellent;.80−.89 good;.70−.79 acceptable (George & Mallery, 2003).*

### 3.5 Data analysis

The analysis was carried out in four phases. First, descriptive statistics were computed to characterize service satisfaction. Second, comparative analysis by area of residence was conducted using design-based Student’s t-tests. Third, a design-based one-way ANOVA with Bonferroni post-hoc tests was applied to compare geographic domains, reporting effect size η². All inferential procedures were implemented using the survey package in R, incorporating expansion factors, strata, and clusters to produce design-consistent standard errors. Fourth, principal component analysis was conducted to explore the dimensional structure of the 21-item Citizen Satisfaction Index using orthogonal Varimax rotation. Component retention was determined using the Kaiser criterion with eigenvalue greater than 1, confirmed by visual inspection of the scree plot. Factor loadings equal to or greater than.40 were considered substantively meaningful. Survey expansion weights were incorporated in all analyses to ensure population-representative estimates. The significance level was α = .05. R v.4.3 was used for data processing, statistical analysis, and visualization, using the survey, psych, and ggplot2 packages. The complete analysis code is available alongside the dataset at https://doi.org/10.6084/m9.figshare.31817545

## 4. Results

### 4.1 Level of satisfaction with public services

The results reveal marked heterogeneity across the 21 public services evaluated. Firefighters and RENIEC obtained almost universal approval with 96.8% and 95.6% respectively, while Social Programs and the Prosecutor’s Office had the lowest levels with 51.9% and 56.4%, evidencing a polarized perception of these critical services. Table 4 presents the level of citizen satisfaction with public services at the national level.

**Table 4:** Level of citizen satisfaction with public services at the national level.

Rk	Public services	n	%VS	%S	%D	%VD	Gap
1	Fire department	27,576	72.9	23.9	2.6	0.5	+93.7
2	RENIEC (National Identity and Civil Registry)	27,447	73.1	22.5	3.5	0.9	+91.3
3	National Police	23,144	50.4	39.3	8.4	1.9	+79.3
4	Comptroller’s Office	21,806	49.5	39.9	8.8	1.7	+78.9
5	Local governments	26,893	46.7	42.5	9.2	1.6	+78.4
6	State offices	26,237	48.7	39.9	9.3	2.1	+77.2
7	SAT (Tax Administration Service)	22,457	46.0	41.9	10.3	1.9	+75.7
8	SUNARP (National Registry of Legal Entities and Natural Persons)	23,978	45.6	41.9	10.4	2.1	+75.0
9	Internet	26,502	46.0	40.4	11.0	2.6	+72.8
10	Public education	24,741	41.1	45.0	11.4	2.6	+72.1
11	Drinking Water	27,217	43.4	42.5	11.3	2.8	+71.8
12	Electricity	27,855	41.6	43.7	11.9	2.8	+70.6
13	Public Health	25,315	37.5	45.6	13.9	3.0	+66.2
14	Ombudsman’s Office	27,891	34.4	48.1	14.5	3.1	+64.9
15	Citizen Security	28,170	40.7	41.0	14.6	3.7	+63.4
16	INDECOPI	23,236	40.2	41.1	15.9	2.8	+62.6
17	Judiciary	24,859	36.2	43.6	16.0	4.2	+59.7
18	Public Transportation	26,923	32.8	37.5	22.3	7.4	+40.6
19	Pension System	27,597	18.7	42.8	30.0	8.5	+23.1
20	Prosecutor’s Office	28,589	27.3	29.1	27.4	16.2	+12.8
21	Social Programs	27,696	16.6	35.3	36.8	11.3	+3.8

Note. VS = Very satisfied. S = Satisfied. D = Dissatisfied. VD = Very dissatisfied. Gap = (VS + S) – (D + VD). Rk = Satisfaction ranking. Source: ENAHO 2024*.*

### 4.2 Satisfaction by area of residence

Significant differences were identified between urban and rural areas in several services. Rural areas show greater satisfaction with public transportation and education, while urban areas exceed rural areas in the pension system. These findings suggest different patterns of perception depending on the territorial context. Table 5 presents the satisfaction levels by area of residence.

**Table 5:** Satisfaction with public services by area of residence.

Public services	% Urban	% Rural	Δ	t	p
Public education	84.9	88.4	−3.5	4.56	<.001***
Public health	81.9	85.4	−3.5	5.61	<.001***
Social programs	50.4	54.4	−4.0	2.14	.032*
Public safety	81.2	82.6	−1.4	6.48	<.001***
Public transportation	67.8	74.8	−7.0	14.77	<.001***
Pension system	63.0	58.9	+4.1	−9.10	<.001***
RENIEC (National Identity and Civil Registry)	95.9	95.1	+0.8	−4.29	<.001***
Drinking water	85.7	86.2	−0.5	1.06	.289
Electricity	85.1	85.6	−0.5	−0.14	.890

Note. % = Percentage of satisfaction (Very satisfied + Satisfied). Δ = Difference between urban and rural areas. t = Student’s t-statistic. *p < .05, ***p < .001.

### 4.3 Regional gaps in citizen satisfaction

Given the cross-sectional nature of this study, the following differences should be interpreted as descriptive associations rather than causal evidence. The ANOVA revealed statistically significant differences between the eight domains, F(7, 27338) = 89.57, p < .001, η² = .022. Bonferroni post-hoc tests identified that the North Coast has the lowest satisfaction index, with significant differences compared to the Southern Highlands, Metropolitan Lima, and Amazon. The Southern Highlands obtained the highest level of satisfaction, even surpassing Metropolitan Lima. Although mean differences are modest in absolute terms, ranging from 3.09 to 3.29 on a 1–4 scale, their substantive relevance lies in their consistency across all 21 services evaluated, systematically disadvantaging populations in regions with documented structural deficits in institutional capacity. Table 6 presents the Citizen Satisfaction Index by geographic domain.

**Table 6:** Citizen Satisfaction Index by geographic area.

Geographic area	n	M	SD	IC 95%	Δ Lima	p
Southern Highlands	3,564	3.288	0.477	[3.273, 3.304]	+0.046	.042*
Central Highlands	4,586	3.276	0.471	[3.262, 3.289]	+0.033	.184
Southern Coast	1,821	3.274	0.532	[3.250, 3.299]	+0.032	.213
Metropolitan Lima	1,886	3.243	0.519	[3.219, 3.266]	(ref.)	—
Central Coast	2,718	3.230	0.486	[3.212, 3.249]	−0.012	.712
Northern Highlands	1,766	3.166	0.545	[3.141, 3.192]	−0.076	.018*
Amazon	6,660	3.127	0.540	[3.114, 3.140]	−0.116	<.001***
Northern Coast	4,345	3.090	0.485	[3.076, 3.104]	−0.153	<.001***

Note. M = Mean, SD = Standard deviation. ISC (range 1–4). ANOVA: F(7, 27338)=89.57, p < .001, η² = .022. *p < .05, ***p < .001 (Bonferroni vs. Metro Lima).

### 4.4 Critical services by geographic area

The North Coast has the lowest average satisfaction rating for the four critical services, with particular deficits in Social Programs and the Public Prosecutor’s Office. In contrast, the Southern Highlands has the highest average, standing out in Public Transportation and the Pension System. Table 7 presents the satisfaction percentages for these critical services broken down by geographic area.

**Table 7:** Percentage of satisfaction with critical services by geographic area.

Domain	Social Programs	Prosecutor’s Office	Pensions	Transportation	Average
Southern Highlands	59.2	56.7	72.0	79.9	66.9
Central Highlands	56.3	60.7	64.1	79.0	65.1
Metropolitan Lima	54.9	57.7	74.3	65.2	63.0
Southern Coast	54.5	58.9	64.0	71.4	62.2
Central Coast	54.4	53.5	69.9	70.4	62.1
Amazon	47.9	61.4	57.9	66.6	58.4
Northern Highlands	49.9	51.7	46.2	68.5	54.1
Northern Coast	43.8	45.5	50.7	61.2	50.3
National	51.9	56.4	61.5	70.3	60.0

*Note: Values expressed as percentage of satisfaction (Very satisfied + Satisfied). Critical services = four with lowest national satisfaction. Domains ranked from highest to lowest average.*

### 4.5 Visualization of regional gaps

**Fig 1:**
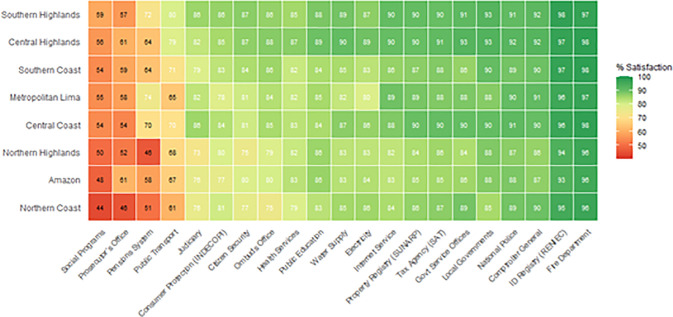
Heat map: Satisfaction with public services and geographical coverage. Note: Color scale: Red indicates low satisfaction; Yellow indicates moderate satisfaction; Green indicates high satisfaction. Rows ordered by Citizen Satisfaction Index. Columns ordered by national satisfaction gap. Source: ENAHO 2024, Governance Module – INEI.

Services such as the Fire Department and RENIEC show uniformly high satisfaction across all domains, while Social Programs and the Prosecutor’s Office show predominantly low satisfaction, particularly in the North Coast and Amazon. Fig 1 presents a heat map showing the percentage of satisfaction for each of the 21 services across the eight geographic domains.

**Fig 2:**
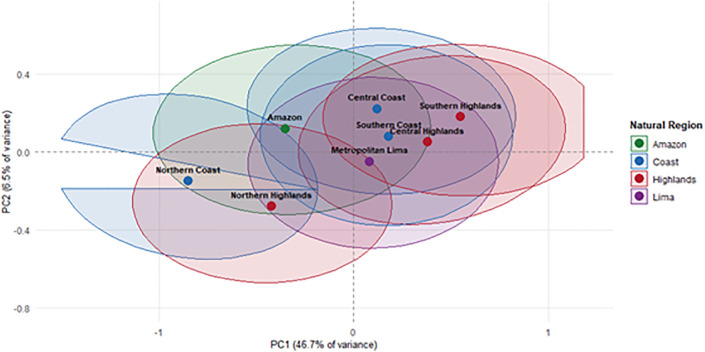
Principal component analysis: Distribution of geographic domains. Note: PC1 explains 46.7% of the variance and represents overall satisfaction. PC2 explains 6.5% of the variance. The ellipses represent 95% confidence intervals by geographic domain. Colors by natural region: Coast (blue), Highlands (red), Amazon (green), Lima (purple). Source: ENAHO 2024, Governance Module – INEI.

Domains positioned to the right of the biplot score higher in overall satisfaction, while those to the left score lower. The 95% confidence ellipses confirm the territorial separation identified in the ANOVA results, with the Southern Highlands clearly separated from the North Coast. Fig 2 presents a biplot from the principal component analysis showing the distribution of geographic domains.

## 5. Discussion

This study provides the first comparative characterization of citizen satisfaction with public services among geographically diverse regions in Peru. The results reveal statistically significant territorial gaps (F = 89.57, p < .001), with the North Coast (M = 3.09) and Amazon (M = 3.13) scoring lower than the Southern Highlands (M = 3.29). While absolute mean differences are modest on the 1–4 scale, their practical significance is better understood through their cross-service consistency, suggesting patterns consistent with structural rather than incidental disparities. This pattern differs from that observed in Asian economies, where Hopland and Kvamsdal [30] found that capitals maintain systematic advantages over peripheral regions. The Peruvian exception may be associated with the greater institutional capacity historically developed in the southern mountain regions, where the consolidated presence of the state and local governance networks facilitate a more effective provision of services than in coastal areas with greater institutional informality. This pattern is consistent with the hypothesis that government trust mediates satisfaction: regions with historically stronger state presence tend to generate higher institutional trust, which in turn elevates satisfaction evaluations even when objective service conditions are comparable. The Southern Highlands, which surpasses Metropolitan Lima in overall satisfaction despite lower per capita income, illustrates this dynamic particularly well [31].

The urban-rural disparities identified show patterns that challenge conventional assumptions. While the prevailing literature documents lower rural satisfaction Zhao et al. [32], the findings show that rural residents in Peru report higher satisfaction with public transportation (+7.0%) and education (+3.5%). Forman-Rabinovici and Beeri [33] and Ochoa-Rico et al. [34] observed similar patterns in Ecuador, attributing them to the expectation disconfirmation model: Rural populations develop expectations calibrated to their territorial reality, favorably evaluating services that meet minimum standards. Services with greater institutional complexity, including social programs (51.9%), the public prosecutor’s office (56.4%), and the pension system (61.5%), simultaneously show the lowest national satisfaction and the largest regional gaps, consistent with Eckhard and Friedrich [35] in that intergovernmental coordination amplifies disparities according to local institutional capacity.

Principal component analysis confirms a multidimensional structure where PC1 (46.7% variance) represents overall satisfaction and clearly differentiates geographical domains. This factorial structure, which groups basic services, justice and security, administrative services, and subnational governments, reflects the institutional architecture of Peru’s decentralized system and coincides with the findings of Núñez-Barriopedro et al. [36] on structures differentiated by sector. The spatial separation between domains in the biplot suggests that territorial gaps transcend specific services, consistent with structural differences in regional institutional capacity. These findings have direct implications for public policy design: while standardized services such as RENIEC (95.6%) and Firefighters (96.8%) achieve homogeneous satisfaction nationally, services requiring intergovernmental coordination demand differentiated interventions according to territorial institutional capacity [37].

The study has limitations that should be considered when interpreting the results. The cross-sectional design prevents establishing causal relationships between institutional capacity and citizen satisfaction. Likewise, the moderate effect size (η² = .022) indicates that variables not included in the analysis explain a substantial portion of the variance in satisfaction. Future research should incorporate objective indicators of local institutional quality to examine its mediating role, as well as longitudinal designs that evaluate the impact of specific reforms. Nevertheless, the findings provide robust evidence on the magnitude and distribution of territorial gaps, constituting a baseline for monitoring policies aimed at reducing inequalities in the provision of public services in decentralized systems with high territorial heterogeneity.

## 6. Conclusion

This study provides the first comparative characterization of citizen satisfaction with public services among geographically diverse regions of Peru, revealing statistically significant territorial gaps that transcend specific services and are consistent with structural differences in institutional capacity. While the overall effect size is modest (η² = .022), indicating that geographic domain explains 2.2% of the variance in satisfaction, the consistent directionality of the patterns across 21 services strengthens their substantive relevance for public policy design [38]. The findings show that the North Coast and Amazon regions consistently experience lower satisfaction than the Southern Highlands and Metropolitan Lima, with particularly pronounced disparities in services that require intergovernmental coordination, such as social programs, the public prosecutor’s office, and the pension system [39]. Urban-rural differences do not follow uniform patterns but vary according to the type of service, suggesting that the model of expectation disconfirmation operates differentially according to territory [40,41]. These results have direct implications for public policy design: while services with nationally standardized protocols achieve homogeneous satisfaction, those dependent on local institutional capacity require differentiated interventions that strengthen territorial governance structures [42], [43]. Future research should incorporate objective indicators of institutional quality and longitudinal designs to assess the impact of specific reforms on reducing territorial gaps, thus contributing to the fulfillment of Sustainable Development Goal 10 on reducing inequalities.

## Supporting information

S1 Table Minimum dataset for replication. Dataset containing the processed variables from the 2024 ENAHO Governance Module used in this study. https://doi.org/10.6084/m9.figshare.31817545.
(XLSX)
